# 

**Published:** 1787

**Authors:** 


### CATALOGUE OF BOOKS.

1. SOME Account of the Walton Water, near Tewkeſbury; with Thoughts on the Uſe and Diſeaſes of the Lymphatic Glands. By *James Johnſtone*, M. D. Phyſician to the General Infirmary, Worceſter; Fellow of the Royal Medical Society, Edinburgh; of the Philoſophical Societies of Mancheſter and Bath, and correſponding Member of the Medical Society, London. 8vo. Worceſter, 1787.

2. Obſervations on the Principles of the old Syſtem of Phyſic, exhibiting a Compend of the new Doctrine: The whole containing a new Account of the State of Medicine from the preſent Times, backwards, to the Reſtoration of of the Grecian Learning in the weſtern Parts of Europe. By a Gentleman converſant in the Subject. 8vo. *Murray*, London, 1787.

3. Obſervations upon the new Opinions of John Hunter, in his late Treatiſe on the Venereal Diſeaſe. By *Jeſſe Foot*, Surgeon. Part the Third. 8vo. *Becket*, London, 1787.

4. Strictures in Vindication of ſome of the Doctrines miſrepreſented by Mr. Foot, in his two Pamphlets, entitled, Obſervations upon the new Opinions of John Hunter, in his late Treatiſe on the Venereal Diſeaſe. By *T. Brand*. 4to. *Nicol*, London, 1787.

5. A Review of Jeſſe Foot's Obſervations on the new Opinions of John Hunter, in his late Treatiſe on the Venereal Diſeaſe. By *Charles-Brandon Trye*. 8vo. *Murray*, London, 1787.

6. An Eſſay on Sea Bathing, and the internal Uſe of Sea Water. By *Richard Kentiſh*, M. D. F. A. S. Edinb. &c. 8vo. *Murray*, London, 1787.

7. An Eſſay on the Method of ſtudying Natural Hiſtory: Being an Oration delivered to the Societas Naturæ Studioſorum at Edinburgh in the Year 1782. By *Richard Kentiſh*, *M. D.* 8vo. *Elmſley*, London, 1787.

8. A ſhort

8. A ſhort Eſſay on the Propagation and Diſperſion of Animals and Vegetables; being chiefly intended as an Anſwer to a Letter lately publiſhed, and ſuppoſed to be written by a Gentleman of Exeter, in favour of equivocal Generation. 12mo. *Wilkie*, London, 1787.

9. Proſpectus of a Syſtem of Anatomy; illuſtrated with above two hundred and forty Copper Plates, collected from the moſt celebrated Authors in Europe. By *Andrew Bell*, F. S. A. S. Folio. London, 1787.

10. A Set of Anatomical Tables, with Explanations, and an Abridgement of the Practice of Midwifery; with a View to illuſtrate a Treatiſe on that Subject, and a Collection of Caſes. By *William Smellie*, M. D. A new Edition, carefully corrected and reviſed, with Notes and Illuſtrations, adapted to the preſent improved Method of Practice. By *A. Hamilton*, M. D. F. R. S. Edin. and Profeſſor of Midwifery in the Univerſity of Edinburgh. 8vo. *Elliot*, London, 1787.

11. Select Caſes in the different Species of Inſanity, Lunacy, or Madneſs; with the Modes of Practice as adopted in the Treatment of each. By *William Perfect*, M. D. 8vo. *Murray*, London, 1787.

12. Obfervations on Poifons, and on the Ufe of Mercury in the Cure of obftinate Dyfenteries. By *Thomas Houlfton*, M. D. late Senior Phyfician to the Liverpool Infirmary, and Honorary Member of the Literary and Philofophical Society of Manchefter, and of the Phyfical Society of Edinburgh. A new Edition, with Additions, Amendments, and an Appendix. 8vo. *Elliot*, Edinburgh, 1787.

13. An Account of the Culture and Ufe of the Mangel Wurzel, or Root of Scarcity. Tranflated from the French of the Abbé *de Commerell*. 8vo. *Dilly*, London, 1787.

14. A concife Account of a new chemical Medicine, entitled Spiritus Æthereus Anodynus, or Anodyne Æthereal Spirit; containing a Relation of its very extraordinary Efficacy in a Variety of Complaints of the moft obftinate and alarming Nature, particularly the Hydrothorax, or Dropfy of the Breaft, and other Species of Dropfy: Alfo of its fpecific Virtue in the Gout and many rheumatic Affections; in hyfterical, hypochondriacal, and various other nervous Complaints, efpecially thofe of the epileptic Kinds; in Afthmas, and all Coughs unattended with Inflammation. With a Word or two, by way of Poftfcript, to Dr. James-

 Mac-

Mackittrick Adair, late of Antigua. By *William Tickell.* 8vo. Bath, 1787.

15. The Families of Plants, with their natural Characters, according to the Number, Figure, Situation, and Proportion of all the Parts of Fructification. Tranflated from the laft Edition (as publifhed by Dr. Reichard) of the Genera Plantarum, and of the Mantiffæ Plantarum of the elder Linneus; and from the Supplementum Plantarum of the younger Linneus; with all the new Families of Plants from Thunberg and l'Heritier. To which is added an accented Catalogue of the Names of Plants, with the Adjectives applied to them, and other botanic Terms, for the Purpofe of teaching their right Pronunciation. By a Botanical Society at Lichfield. 2 Vols. 8vo. Lichfield, 1787.

16. Reports of the Humane Society, inftituted in the Year 1774, for the Recovery of Perfons apparently drowned. For the Years 1785 and 1786. 8vo. *Dodfley*, London, 1787.

17. Differtatio Medica Inauguralis de Corporum humanorum temperamentis, morbifque nonnullis quibus horum quidque maxime pendeat. Auctore *Joanne Ainflie*, Scoto. 8vo. Edin. 1787.

18. Dif-

18. Dissertatio Medica Inauguralis de Diarrhœa. Auctore *Campbel Betham*, Scoto. 8vo. Edin. 1787.

19. Dissertatio Medica Inauguralis de Asthmate periodico. Auctore *Andr. Carrick*, Britanno. 8vo. Edin. 1787.

20. Tentamen physiologico-medicum inaugurale de Secretione uterina, vel fluxu qui vulgo menstruus dicitur. Auctore *Joanne Craven*, Hiberno. 8vo. Edin. 1787.

21. Tentamen Medicum Inaugurale de Insania. Auctore *Francisco Duncan*, Scoto. 8vo. Edin. 1787.

22. Tentamen Medicum Inaugurale de ista Herniæ uterinæ Specie quæ Retroversio Uteri vulgo dicitur. Auctore *Thoma Gill*, Anglo. 8vo. Edin. 1787.

23. Dissertatio Medica Inauguralis de Rabie Canina. Auctore *Jacobo M'Ilwaine*, Hiberno. 8vo. Edin. 1787.

24. Tentamen Medicum Inaugurale de Febre puerperarum. Auctore *Alexandro Jackson*, Hiberno. 8vo. Edin. 1787.

25. Dissertatio Medica Inauguralis de Pneumonia. Auctore *Carolo Kerr*, Britanno. 8vo. Edin. 1787.

26. Tentamen Medicum Inaugurale quædam complectens de Morbis ex graviditate pendentibus. Auctore *Gulielmo Leeky*, Hiberno. 8vo. Edin. 1787.

27. Tractatus Inauguralis de Febre remittente Marilandica. Auctore *Daniel Moores*, reipublicæ Marilandicæ Cive. 8vo. Edin. 1787.

28. Tentamen Medicum Inaugurale quædam complectens de Typho. Auctore *Thoma Renwick*. 8vo. Edin. 1787.

29. Dissertatio Medica Inauguralis de Leucorrhœa. Auctore *Joanne Simpson*, Hiberno. 8vo. Edin. 1787.

30. Dissertatio Medica Inauguralis de Medicina Sectæ Methodicæ veteris. Auctore *Thoma Smith*, Dunelmensi. 8vo. Edin. 1787.

31. Dissertatio Medica Inauguralis de Apoplexia. Auctore *Carolo Stewart*, Britanno. 8vo. Edin. 1787.

32. Dissertatio Medica Inauguralis de Cynanche Maligna. Auctore *Roberto Walker*, Virginiensi. 8vo. Edin. 1787.

33. Dissertatio Medica Inauguralis de Rachitide. Auctore *Joanne-Watson Sproule*, Hiberno. 8vo. Edin. 1787.

34. *Josephi Emmanuel de Davalos*, Limani apud Peruvianos, Specimen Academicum de morbis morbis Limæ graſſantibus, ipſorumque Therapeia. 8vo. Monſpelii, 1787.

35. Diſſertatio Medica de morbis epidemicis. Auctore *Joanne-Bened. Zandyck*, M. D. 4to. Douay, 1786.

36. Fundamenta Botanica, ſive Philoſophiæ Botanicæ explicatio a *Domenico Cirillo*, M. D. 8vo. Neapoli, 1786.

37. *Johannis Meurſii* de Puerperio Syntagma; cum Hiſtoria monſtroſæ Partium genitalium Conformationis in Adulescente, Animadverſionibus illuſtrata, Edidit *Joh. Georg Frid. Franzius*. 8vo. Lipſiæ, 1785.

38. De Sanguine et de Sanguineis Concretionibus per Anatomen indagatis, et pro Cauſis Morborum habitis, Quæſtiones Medicæ. Auctore *Joſepho Paſta*, Bergomate, in Patria Protophyſico, Noſocomii majoris Medico. 8vo. Bergoma, 1786.

39. *Joannis Brugnoni*, Chir. Colleg. Direct. Reg. Schol. veter. de Teſtium in Fœtu poſitu; de eorum in Scrotum deſcenſu; de tunicarum, quibus hi continentur, numero et origine Diſſertatio. 4to. Auguſt. Taurin. 1786.

40. *Franciſci-Henrici Birnſtiel*, M. D Civitatis Bruchſalienſis atque in eadem Copiarum militarium, Noſocomii F. Miſericordiæ ad Sanctum Lazarum, Orphanotrophii et Sophronisterii, et Principatus Spirensis Cis-rhenani, Physici ac Medici, de Dysenteria Liber, sistens, præter completam Dysenteriarum in Annis 1778, 1779, et 1780, epidemicarum Historiam, hujus Morbi singularem Naturam, Causam, et Hippocraticam medendi Methodum, unà cum perbrevi morborum intercurrentium recensione. 8vo. Manheim, 1786.

41. De Medicis veterum Hebræorum eorumque Methodo sanandi Morbos pauca disserit *Jo. Henr. Lautenschlager*. 4to. Schleiz, 1786.

42. Histoire d'une Symphyseotomie, pratiquée avec Succés pour la mere et pour l'Enfant, le 23 Janvier, 1786; par M. *Verdier du Clos*, Docteur en Medecine de l'Université de Nancy, Correspondant de la Société Royale de Medecine de Paris, &c. 8vo. Mans, 1787.

The subject of this history was a woman of low stature, and twenty-nine years old, who had been ricketty from her infancy, and was in a bad state of health. Her labour pains commenced on the 20th of January in the morning, and the section of the symphysis was performed by M. Verdier on the 23d in the evening. The bones are said to have separated to the the diſtance of two inches and ſix tenths, and the child, we are told, was eaſily delivered, but died within an hour after its birth. The mother recovered, and, at the end of two months, the bones of the pubis were completely reunited, and ſhe was able to walk and go about her buſineſs as uſual.

43. Extrait des Regiſtres de l'Academie Royale des Sciences, du 20 Juin, 1787. Rapport des Commiſſaires chargés, par l'Academie, des Projets relatifs à l'etabliſſement des quatres Hopitaux. 4to. Paris, 1787.

44. Memoire ſur les Maladies les plus frequentes a Grenoble; ſuivi d'un Eſſai ſur la Topographie de cette ville. Par M. *Villars*, Medecin de l'Hopital et de celui de la Charité, Correſpondant de la Société Royale de Medecine de Paris, &c. 8vo. Grenoble, 1787.

45. Memoire ſur les Maladies les plus familieres a Rochefort; avec des Obſervations ſur les Maladies qui ont regné dans l'Armée Navale combinée pendant la Campagne de 1779. Par M. *Lucadou*, Medecin de la Marine dans ce Departement, et chargé des Fonctions de premier Medecin dans cette Armée. 8vo. Paris, 1787.

46. Traité des Maladies Veneriennes. Par M. *Jean Hunter*, des Sociétés Royales des Sciences de Londres et de Gothemburg, Associé Etranger de la Société Royale de Medecine, et de l'Academie Royale de Chirurgie de Paris, Chirurgien extraordinaire de S. M. Britannique, Chirurgien General en second des Forces de Terre de la Grande Bretagne, et de l'Hopital de Saint George. Traduit de l'Anglois, par M. *Audiberti*, Docteur en Medecine, Correspondant des Academies Royales des Sciences de Turin, et de Chirurgie de Paris, Membre du College Royal de Chirurgie de Turin, et Chirurgien Major du Regiment Suisse Valaisan de Courtan, au Service de S. M. le Roi de Sardaigne. 8vo. Paris, 1787.

47. Physikalische chemische Versuche und Beobachtungen. *i. e.* Physico-chemical Experiments and Observations. By *Sigisbert Fred. Hermstadt*. Part I. 8vo. Berlin, 1787.

48. Neue Versuche zu einer wahren Physiologie der Galle. *i. e.* New Experiments for an accurate Physiology of the Bile. By *Sebastian Goldwiz*, M. D. 8vo. Bamberg, 1785.

